# Tunable recombinant protein expression in *E. coli*: enabler for continuous processing?

**DOI:** 10.1007/s00253-016-7550-4

**Published:** 2016-05-12

**Authors:** Lukas Marschall, Patrick Sagmeister, Christoph Herwig

**Affiliations:** Institute of Chemical Engineering, Research Area Biochemical Engineering, Vienna University of Technology, Vienna, Austria; Exputec GmbH, Vienna, Austria; Christian Doppler Laboratory for Mechanistic and Physiological Methods for Improved Bioprocesses, Vienna University of Technology, Gumpendorferstraße 1a/166-4, 1060 Vienna, Austria

**Keywords:** All-or-none induction, Continuous processing, *E. coli*, Transcription, Tunable

## Abstract

Tuning of transcription is a powerful process technological tool for efficient recombinant protein production in *Escherichia coli*. Many challenges such as product toxicity, formation of inclusion bodies, cell death, and metabolic burden are associated with non-suitable (too high or too low) levels of recombinant protein expression. Tunable expression systems allow adjusting the recombinant protein expression using process technological means. This enables to exploit the cell’s metabolic capacities to a maximum. Within this article, we review genetic and process technological aspects of tunable expression systems in *E. coli*, providing a roadmap for the industrial exploitation of the reviewed technologies. We attempt to differentiate the term “expression tuning” from its inflationary use by providing a concise definition and highlight interesting fields of application for this versatile new technology. Dependent on the type of inducer (metabolizable or non-metabolizable), different process strategies are required in order to achieve tuning. To fully profit from the benefits of tunable systems, an independent control of growth rate and expression rate is indispensable. Being able to tackle problems such as long-term culture stability and constant product quality expression tuning is a promising enabler for continuous processing in biopharmaceutical production.

## Introduction

The relevance of the gram-negative bacterium *Escherichia coli* for the basic biotechnological research as well as for industrial exploitation is outstanding. *E. coli* served as the primary model organism within the development of modern biotechnology. As a consequence, researchers today have access to a broad spectrum of genetic tools and cultivation techniques, enabling simple and predictable genetic manipulation and cultivation on inexpensive media to high cell densities. As regards industrial exploitation, *E. coli* emerged as the primary production workhorse for the biotechnological production of primary and secondary metabolites as well as recombinant proteins. This is reflected by the fact that 29 % of all biopharmaceutical products approved as biopharmaceuticals between 2010 and July 2014 are produced in *E. coli* (Walsh 2014).

Overall productivity and product quality obtained from *E. coli* processes is determined by the complex interplay of processing mode and the product to be produced as well as the expression system applied.

The main challenges of recombinant protein production in *E. coli* are associated with non-suitable (too high or too low) level of recombinant expression:

1. First, high-level expression and the presence of foreign plasmids drain the hosts’ metabolic resources (often referred to as metabolic load or metabolic burden) (Bentley et al. 2009; Bienick et al. 2014; Glick 1995; Mairhofer et al. 2013). Metabolic load often resulting in depletion of amino acids or aminoacyl-tRNAs and triggering heat-shock response can therefore ultimately affect product quality-related issues (specific activity, stability, and immunogenicity) and product quantity-related issues (product degradation, lower specific product yields, lower biomass yields, or shorter culture stability).
2. Second, high-level expression of recombinant products can lead to the formation of unfolded or partially folded insoluble protein aggregates known as inclusion bodies which show no catalytic function or activity (Baig et al. 2014; Kane and Hartley 1991).
3. Third, the production of many recombinant products, especially proteins containing disulfide bridges, demands translocation between compartments of the *E. coli* cell factory (Baneyx and Mujacic 2004). Here, too high levels of recombinant protein expression can lead to the blocking of translocation pathways.

**Fig. 1:**
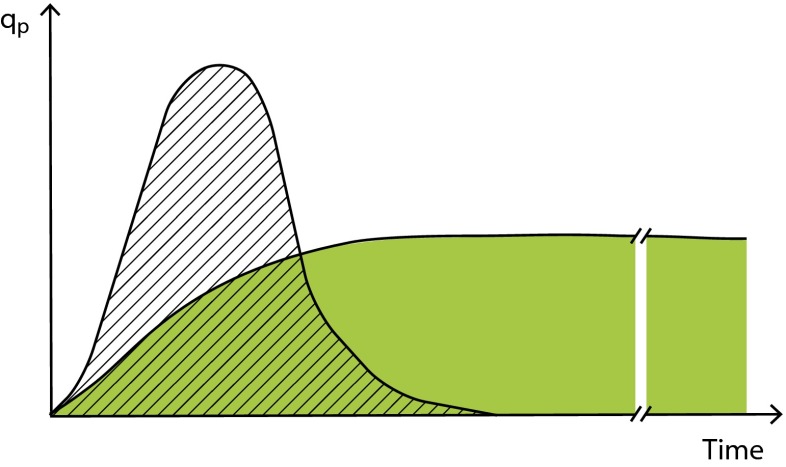
By reducing the protein expression level, the cells can be maintained in a productive state for a longer time, which results in higher end product titers (*q*_*p*_ specific cellular productivity)

The mentioned challenges are either fully or to a great extent caused by recombinant protein expression. The level of recombinant protein expression is affected by the strength of the expression system which involves the strength of the promoter used and the plasmid copy number (Keasling 1999) as well as the process technological parameters such as temperature and the specific growth rate (Hellmuth et al. 1994; Rodríguez-Carmona et al. 2012). It is frequently observed that a reduction of the protein expression level leads to increased end product titers, since the cells can be maintained in a productive state for a longer time (Sagmeister et al. 2014; Sagmeister et al. 2013b) (Fig. 1).

“Expression tuning,” also referred to as “fine-tuning of protein production” and “modulation of expression,” intends to solve the aforementioned challenges by providing a technological framework to adjust recombinant protein expression online to a level which is optimal for protein folding, protein translocation, and long-term productivity. Hence, tuning allows exploiting the cell factory to a maximum.

To our knowledge, expression tuning has not been reviewed so far. With this review article, we are the first to aim at giving a concise and comprehensive overview of current state-of-the-art methods and technologies for expression tuning. First, we briefly discuss the role of continuous processing in the production of biopharmaceuticals and the benefits of its application in combination with expression tuning. Second, we discuss the contemporary scientific conception of expression tuning and aim at the proposition of a sound and comprehensive definition. Third, we review and discuss methods for expression tuning. Special attention is drawn on features relevant to enable expression tuning on cellular level. Furthermore, their integration to process technological methods to achieve expression tuning is discussed. Fourth, we propose a roadmap for the development of industrial tunable expression systems. Finally, in an outlook chapter, we discuss the applicability and benefits of tunable expression systems for more efficient bioprocessing and the acceleration of bioprocess development.

## Tuning—paving the way for continuous processing in biopharmaceutical industry

While continuous manufacturing is widely applied across industries including the petrochemical, food, and pharmaceutical sectors, it is still outnumbered by batch and semi-batch processes in the biopharmaceutical industry (Konstantinov and Cooney 2014). Though downstream and product formulation unit operation already use continuous processing, it is hardly employed in upstream processing. In addition to logistic barriers like challenging implementation and validation complexity, continuous processing suffered from process inherent obstacles like culture instability, lack of process control, and sterility issues (Farid et al. 2014; Stock et al. 2014). However, the demand for flexible manufacturing facilities and reducing cost of goods is increasing due to market fluctuations and growing competition from biosimilars (Kelley 2009; Stock et al. 2014; Walsh 2014; Warikoo et al. 2012). With new technologies emerging in the course of the PAT initiative, the upswing of continuous processing is also welcomed and supported by regulatory authorities (Lee et al. 2015; Myerson et al. 2015). From a regulatory or process technological point of view, many obstacles did decrease or vanish. However, culture stability is still an issue (Nancib and Boudrant 1992). By reducing the protein expression level, cells can be longer maintained in a producing state. Expression tuning can therefore offer a great benefit and act as an enabling tool for continuous processing. In contrast to just using a low producing mutant strain, a tunable host offers the possibility to vary the protein expression online and therefore provides a novel degree of freedom to maneuver an out-of-specs process, back into the design space.

## Defining tunable recombinant protein expression

Although many authors refer to “tunable recombinant protein expression” for various purposes, to our knowledge, a clear and uniform definition is still missing in literature. Some authors refer to “tuning” of expression or gene dosage as adjusting the plasmid copy number (Camps 2010; Xu et al. 2013), which can also unintentionally be submitted to change in course of the bioprocess (Teich et al. 1998). Another definition of tuning of expression refers to the modulation of promoter strength by construction of a set of promoters of different strengths through promoter engineering (Alper et al. 2005; Brewster et al. 2012; Dehli et al. 2012; Mey et al. 2007). In these cases, the actual tuning is achieved through genetic engineering. Furthermore, the adjustment of the expression level via simple process parameters such as temperature and medium composition is sometimes termed expression tuning as well (Correa and Oppezzo 2011). Tuning is often referred to as adjusting the production of recombinant proteins on cellular level, whereby tuning solely on population level (e.g., the formation of subpopulations) is clearly excluded from the definition (Khlebnikov et al. 2002; Lee and Keasling 2005; Striedner et al. 2003). However, this definition only considers the tuning with respect to specific titer and not the specific cellular productivity (*q*_p_), which is varying along the production period (Sagmeister et al. 2014). When speaking of tuning of *q*_p_, one has to bear in mind that this definition does not consider the location of the bottlenecks for recombinant protein production, whereby either transcription, translation, translocation, or folding of recombinant proteins can be bottlenecking (Baneyx and Mujacic 2004; Brinkmann et al. 1989; Harris and Kilby 2014; Tegel et al. 2011; Wagner et al. 2007) (Eq. 1). Keeping in mind that the effective specific cellular productivity (*q*_p_eff_) is actually composed of *q*_p_ and the product degradation rate (*q*_degradation_) (Eq. 2), we propose the following definition for expression tuning:

Expression tuning is referred to as the purposeful adjustment of the recombinant gene transcription rate on cellular level.

1 $$ {q}_{\mathrm{p}}= \min \left({q}_{\mathrm{transcription}},{q}_{\mathrm{translation}},\left({q}_{\mathrm{translocation}},\right){q}_{\mathrm{folding}}\right) $$

2 $$ {q}_{\mathrm{p}\_\mathrm{eff}}={q}_{\mathrm{p}}-{q}_{\mathrm{degradation}} $$

## Formation of subpopulations of producing and non-producing cells (bistable behavior)—an impediment to expression tuning

Following the presented definition of expression tuning, it is of utmost importance to understand, consider, and make it impossible that observed tuning on population level might be attributed to the formation of subpopulations of producing and non-producing cells. If this is the case, tuning of the expression levels is not achieved, which is also called “bistable behavior,” referring to the formation of two subpopulations. Homogeneous expression on cellular level is also often termed “graded response.” Here, knowledge on the mechanisms that lead to these “all-or-none induction phenomena” is reviewed.

Novick and Weiner (1957) identified for the lac operon that apparent tuning on population level may be the result of the formation of subpopulations of fully induced and non-induced cells (all-or-none induction, bistability). This was confirmed by Maloney and Rotman (1973). They observed that the inducer amount only influenced the number of induced cells rather than the transcription rate on cellular level and proposed a model describing this phenomenon (Novick and Weiner 1957). Forty years later, Siegele and Hu (1997) reported this behavior for the arabinose utilization pathway. Further studies using mechanistic modeling and single cell analytics investigated autocatalytic systems and the switching kinetics of inducible systems (Carrier and Keasling 1999; Fritz et al. 2014; Megerle et al. 2008; Ozbudak et al. 2004). Afroz et al. (2014b) investigated and compared eight metabolic pathways of *E. coli* with respect to all-or-none behavior and created a deterministic model. Their model related the type of response (graded or bistable) to the strength of positive feedback (inducible inducer transport) and negative feedback (inducible inducer catabolism). Bistable responses were expected for low negative feedback and high positive feedback and graded responses for high negative feedback and low positive feedback. The extent of bistability of different pathways is believed to be influenced by cooperativity in the expression of pathway transporters (Afroz et al. 2014b).

## General strategies to achieve tuning

Based on the insights in the mechanistic that lead to unwanted bistable behavior (formation of subpopulations of producing and non-producing cells), several strategies based on targeted engineering of metabolic pathways have been reported.

Several authors perform knockout of genes for inducer transport to omit bistable responses (Afroz et al. 2014b; Khlebnikov et al. 2000; Marbach and Bettenbrock 2012). The knockout of transport genes breaks the positive feedback of the inducer on the production of its own transport proteins and eliminates the bistable behavior (Afroz et al. 2014a; Fritz et al. 2014; Khlebnikov et al. 2000). Using inducer transport knockout strains, induction can be achieved by induction with gratuitous inducers (Marbach and Bettenbrock 2012) or by induction with the natural inducer. While some gratuitous inducers can enter the cell passively by diffusing through the membrane, some natural inducers have to be actively transported into it. Introducing a separate copy of the transporter gene under control of a different promoter enables inducer to be transported into the cell while avoiding the positive feedback (Afroz et al. 2014a; Khlebnikov et al. 2000).

In another approach Wagner et al. constructed a strain, termed Lemo21(DE3), where the widely used BL21(DE3) (Studier and Moffatt 1986) was modified by addition of a vector harboring T7 lysozyme under control of the *rhaBAD* promoter. Modulation of T7 lysozyme expression, a natural inhibitor of T7 polymerase, by varying the rhamnose concentration was reported to convert the all-or-none response of the BL21(DE3) strain to a uniform response (Schlegel et al. 2012; Wagner et al. 2008).

We recently showed for the araBAD operon that the use of metabolizable inducer in a mixed-feed environment results in a graded response (Sagmeister et al. 2013b). By using an *E. coli* strain with intact arabinose operon, we were able to tune protein expression with arabinose as metabolizable inducer. Using this strategy, the recombinant protein expression can be tuned by simply adjusting the uptake rate of the inducing substrate.

## Process technological aspects of expression tuning

To our knowledge, expression tuning has so far only found little application in studies using bioreactors and even only in fed-batch mode (Sagmeister et al. 2013b; Striedner et al. 2003). As far as we know, it has so far not been applied in continuous processes. When applying expression tuning, the induction mode is of utmost importance. Within this section, we review several so far used induction strategies and discuss them with respect to expression tuning.

One-point addition of varying non-metabolizable inducer concentrations is the most commonly applied method to achieve expression tuning (Khlebnikov et al. 2002; Lee and Keasling 2005; Wagner et al. 2008). This is typically used for investigative studies with small-scale experiments and short production periods. It is usually assumed that the concentration of inducer per cell (specific inducer concentration) is constant. While this assumption is justifiable for small-scale shake flask experiments, it definitely does not hold true for industrial fermentation processes. Here, the specific concentration of active inducer can be submitted to change due to catabolism, dilution by growth and inactivation, for example, through acetylation (Marbach and Bettenbrock 2012; Novick and Weiner 1957).

**Fig. 2:**
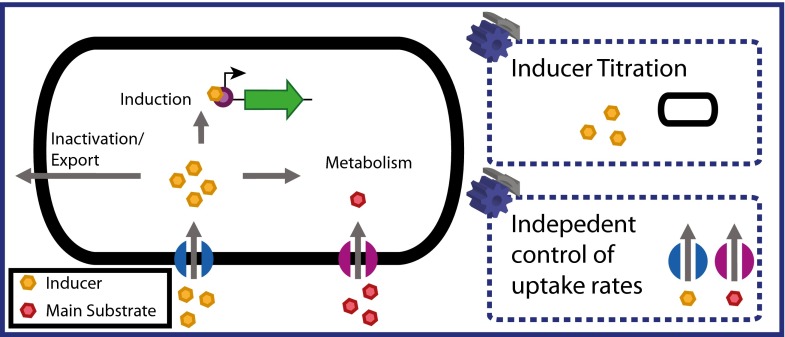
In order to maintain a constant expression level under subsaturating inducer concentrations, a constant inducer concentration within the cell is mandatory. Therefore, all routes of transport into (active import, diffusion) and out of the cell (metabolism, inactivation, export) have to be considered. As not all of these rates are accessible, a constant inducer concentration within the cell can only be approximated. When using non-metabolizable inducers, this condition can be approximated by adjusting the extracellular inducer concentration to the biomass concentration. In the case of metabolizable inducers, a mixed-feeding strategy, where the uptake of the main substrate and inducing substrate can be controlled independently, needs to be applied in order to retain the additional degree of freedom of expression tuning

When exploiting tunable systems for industrial bioprocesses with extended production phase, a continuous inducer supply is necessary to ensure a constant inducer concentration within the cell (Fig. 2). As this criterion is hard to meet, a possible simplification is to neglect the inducer consumption and transporting rates in and out of the cell and simply adjust the inducer amount to the biomass concentration. A method that compensates for these effects, is the continuous feeding of inducer in order to achieve a constant inducer-to-biomass ratio (Striedner et al. 2003), which requires a continuous estimate of biomass concentration. A possible way to achieve that is to use an exponential feeding profile and adjust the inducer concentration to the calculated value according to the feeding profile. These feed-forward strategies assume a constant yield of biomass on substrate (*Y*_*x*/*s*_) and neglect the fact that *Y*_*x*/*s*_ can change especially in production phases. Therefore, a more accurate method is to estimate the biomass based on online accessible data with soft sensors (Luttmann et al. 2012; Paulsson et al. 2014; Sagmeister et al. 2013c; Wechselberger et al. 2013). The type of inducer (metabolizable or non-metabolizable) results in several consequences with respect to the controllability of the system. When using a non-metabolizable inducer, the induction rate can be independently controlled from the substrate uptake rate (or respectively the specific growth rate). When using metabolizable inducers as sole carbon source, the induction rate is tightly coupled to the sugar uptake rate and therefore cannot be controlled independently.

Another alternative is the application of a mixed-feeding strategy, where multiple carbon sources are fed to the cells in certain ratios. The mixed-feed strategy permits the use of metabolizable inducer while retaining the advantage of independent control of induction rate and substrate uptake rate (which determines the specific growth rate). Recently, we demonstrated the tunability of a system via adjusting the uptake rates of two sugars: one acting as carbon source and the other as inducer and second carbon source (Sagmeister et al. 2013b). Independent control of sugar uptake rates can be achieved via generic control strategies for fed-batch processes (Sagmeister et al. 2013c). For mixed-feed systems, catabolite repression poses a natural limitation to the application of these systems, which has to be investigated beforehand and considered for process design (Sagmeister et al. 2013a).

However, when adjusting the inducer amount solely to the cell concentration, the change in the cells’ metabolic load caused by protein expression is not accounted for. In order to do so, the metabolic load needs to be quantified. A possible solution for this matter was provided by Kraft et al. (2007). They created a reporter system based on the firefly luciferase gene *lucA* under the control of a σ^32^-dependent promoter. The heat-shock response caused by formation of mis-folded proteins and aggregates led to expression of luciferase, showing a linear dependency. Higher amounts of mis-folded proteins led to higher luminescence values (Kraft et al. 2007). By rendering the metabolic load accessible to quantification, such reporter systems could allow to use tunable systems in a more accurate way. However, to our knowledge, feedback control of protein expression based on the metabolic load of the host has not been performed so far.

**Fig. 3:**
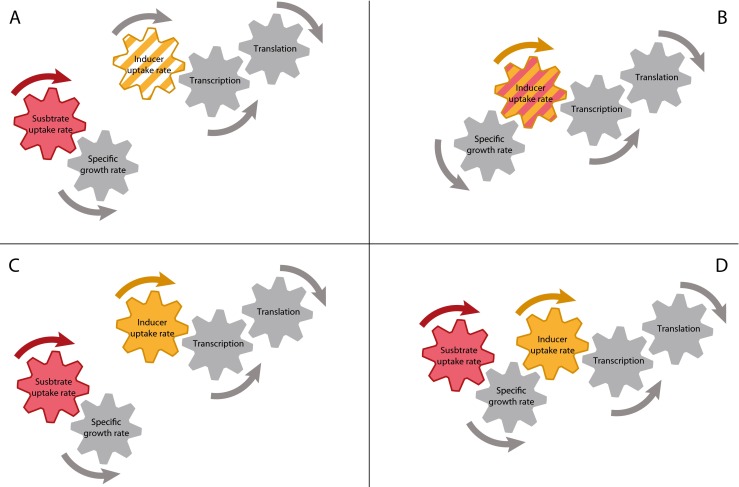
For expression tuning, several induction strategies have been used. One-point addition of non-metabolizable inducer (**a**) is commonly applied, but provides no novel degree of freedom for process control. The inducer concentration is only added at the timepoint of induction and not adjusted throughout the process. It is therefore submitted to change due to catabolism, dilution by growth, and inactivation. By inducer titration, the inducer concentration is continuously added to the culture and concentration changes can therefore be accounted for. When using a metabolizable inducer for inducer titration (**b**), the specific growth rate and the protein expression cannot be controlled individually as the inducer acts as substrate as well. Independent control of specific growth rate and protein expression rate and therefore a novel degree for process control is gained when performing inducer titration with non-metabolizable inducers (**c**). In this case, addition of substrate controls the specific growth rate and addition of inducer controls the induction rate. Another way to retain this novel degree of freedom is to use metabolizable inducers in a mixed-feed environment (**d**). Here, specific growth rate is controlled by addition of main substrate and inducing substrate and induction rate is controlled by the ratio of inducing substrate to main substrate. For reasons of easier comprehensibility, the overall influence of specific growth rate on protein expression is not illustrated in this graphic representation

As described, expression tuning has been technologically implemented in various ways, which significantly differ in the degree of freedoms they leave with respect to process control. Two methods have to be highlighted for providing the highest degree of freedom while containing full controllability of the process: inducer titration with gratuitous inducer and mixed-feeding strategy with a metabolizable inducer as second carbon source. Both methods enable the independent control of specific growth rate and induction rate (Fig. 3).

A short outline of currently applied induction methods is given in Table 1.

**Table 1:** Process technological methods for expression tuning

Induction method	Mode of action	Comments
One-point addition of inducer	The concentration of inducer is adjusted to a defined concentration in the beginning of the induction phase	Widely applied, however, inducer concentration can change throughout the process due to catabolism, dilution by growth, or inactivation (Marbach and Bettenbrock 2012; Novick and Weiner 1957)
Inducer titration	Non-metabolizable inducer is continuously supplied to keep the specific inducer concentration (inducer/biomass) constant	Independent control of sugar uptake rate (growth rate) and induction rate is possible (Striedner et al. 2003)
Metabolizable inducer	Metabolizable inducer is continuously supplied and metabolized by the cells	Induction rate and uptake rate of metabolizable inducer are tightly coupled
Mixed feed	Both primary growth substrate and metabolizable inducer are simultaneously supplied and metabolized by the cells	Independent control of specific growth rate and induction rate is possible (Sagmeister et al. 2013a).

## How to apply tuning

### Verification and evaluation of expression tuning

With respect to the definition as the adjustment of the recombinant gene transcription rate on cellular level, several things have to be considered in order to demonstrate expression tuning. First of all, culture homogeneity needs to be verified in order not to mistake tuning on population level with tuning on cellular level. The use of a fluorescence reporter protein with suitable analytical methods has now established itself in order to prove expression on cellular level. Where flow cytometry (Khlebnikov et al. 2002; Lee and Keasling 2005; Sagmeister et al. 2013b; Wagner et al. 2008) is more commonly used for bioprocesses, microscopy is rather used for investigational studies on induction kinetics and behavior (Megerle et al. 2008; Ozbudak et al. 2004; Siegele and Hu 1997). Other important aspects have to be addressed when investigating bioprocesses: outgrowth of segregants, where non-producing cells outgrow producing cells, can be caused by loss of plasmids (Krone et al. 2007; Smith and Bidochka 1998) or all-or-none induction (Novick and Weiner 1957). The transcription rate of the used promoter might change during the process according to its response time (Lee and Keasling 2005). Regarding these time-dependent effects, it is necessary to gather time-resolved data to be able to attribute the observed responses to the right causes. After verification of the tunable system, its performance needs to be evaluated. In order to gain physiological knowledge, it is also well established to compare specific concentrations (related to biomass concentration or cell number) (Khlebnikov et al. 2002; Lee and Keasling 2005) rather than volumetric concentrations.

Furthermore, it needs to be considered that the specific cellular productivity (*q*_p_) is varying along the production period (Sagmeister et al. 2014). This behavior is not reflected if solely end-point-specific concentrations are monitored.

In order to define comparable criteria when working with tunable systems we recommend the following strategy:

1. Use of fluorescent reporter protein and suitable analytical methods (flow cytometry or microscopy)
2. Time-resolved acquisition of product data
3. Specific cellular productivity as target variable

Considering the maturity of tunable expression technologies, we anticipate that in the future, more studies will focus on the investigation and characterization of tunable systems in industrially relevant fermentation processes.

### Prerequisites for expression tuning on cellular level

From the reviewed literature, general concepts for prerequisites for expression tuning on cellular level can be abstracted. Above all, a tunable promoter system is the fundamental requirement for expression tuning. For better control, it is important to maintain a constant inducer concentration within the cell. When designing inducible systems, it is therefore necessary to consider all possible routes for inducer concentration changes within the cell such as transport in and out of the cell, assimilation, and change of the specific inducer concentration due to cell growth. A large dynamic range of tunability with respect to inducer concentrations is of advantage. Furthermore, plasmid stability is necessary. Otherwise, the transcription of each gene copy is controlled, but protein expression per cell varies due to different amounts of plasmid copy number and results in an inhomogeneous culture (Khlebnikov et al. 2000). Therefore, the use and proof of stable plasmids or genome integration of the expression sequence is mandatory. Despite of its stability, the plasmid copy number of the used vector itself needs to be considered. Whether the use of a low-, medium- or high-copy number plasmid is possible, depends on the strength of the used tunable promoter system. The direct controllability of protein expression by transcription tuning is only possible as long as transcription is the limiting step for recombinant protein production. It might happen that a weak promoter on a high-copy number plasmid already exceeds these limits and other steps rather than transcription become the bottleneck in recombinant protein production. This loss of controllability can finally lead to a decrease of product quality (aggregation, amino acid incorporation, etc.) (Harris and Kilby 2014; Kane and Hartley 1991) or lead to a loss of plasmids (Chang et al. 2003). In order to receive more controllability, the tightness of the used expression system needs to be taken into consideration. Basal product expression can sometimes be a major issue as it is for, e.g., membrane protein production (Giacalone et al. 2006; Wagner et al. 2007).

Other prerequisites valid for “the ideal expression system” also apply to tunable expression systems and are discussed elsewhere (Keasling 1999; Rosano and Ceccarelli 2014).

### Expression tuning also an opportunity for process development and scientific progress

Combined with process technology, tunable expression systems enable the control of the recombinant protein expression rate using process technological means. With the use of a suitable control strategy, the transcription rate can be indirectly controlled. Subsequently, this can be considered in process development and process optimization, for example, using design-of-experiment approaches (Mandenius and Brundin 2008). Expression tuning using process technological means adds a novel degree of freedom to the design of recombinant processes. Using the reviewed technologies, it is possible to fine-tune recombinant protein expression to a level which exploits the cell factory to a maximum. This optimization can take place in controlled lab-scale bioreactor experiments, which more accurately reflects industrial processes that screening studies in shake flasks.

In the field of enzyme control analysis, it is necessary to use promoters of different strengths in order to investigate different molecular fluxes within the cell (Jensen et al. 1993). This approach involves the construction of different constructs for different concentrations of observed enzymes. We anticipate that the construction of only one tunable construct to cover all cases would be a great benefit for the investigation and optimization of metabolic pathways. Possible fields of applications for expression tuning are summarized in Table 2.

**Table 2:** Use cases for expression tuning

Use case	Mode of action	Reference
Increase soluble protein titer	Prevent unwanted inclusion body formation through downregulation of expression	(Baig et al. 2014; Kane and Hartley 1991)
Increase active product amount	Debottleneck translocation for correct disulfide bond formation in periplasm	(Baneyx and Mujacic 2004)
Toxic protein expression	Tune toxic protein expression to a level which is tolerated by the host	(Doherty et al. 1993; Dong et al. 1995)
Reduce metabolic load	Enable longer production periods and increase product quality by lowering the burden on the host and its protein expression machinery	(Bentley et al. 2009; Bienick et al. 2014; Glick 1995; Mairhofer et al. 2013)
Substitute and supplement to promoter libraries	Facilitate enzyme control analysis by using a tunable promoter instead of several promoters of different strengths	(Alper et al. 2005; Dehli et al. 2012; Mey et al. 2007)

## Conclusions and outlook

Within this contribution, we provide an overview of challenges and applications of state-of-the-art methods and technologies for expression tuning in *E. coli*. Furthermore, we attempt to provide a clear and precise definition for expression tuning: as the etymology of tuning meets more the conception of stepping on the accelerator pedal than on building a whole new car in order to make your car go faster, we anticipate to use the term tuning as the purposeful adjustment of the recombinant gene transcription rate on cellular level.

To date, one-point addition of inducer is the most prevailing process technological method to achieve expression tuning. However, due to inherent problems such as degradation of inducer and a lack of process technological control over the tuning process, we anticipate that other process technological methods such as the use of metabolizable inducers, inducer titration, and mixed-feed strategies will gain importance in the future. We anticipate that expression tuning will unfold its full benefits only in combination with adequate control strategies. Hence, these methods will be essential for the industrial exploitation of tunable expression systems.

Considering the broad spectrum of mature methods and technologies as well as the broad scientific knowledge available, we anticipate that expression tuning will soon be adapted by industry as generically applicable tool to enable and optimize the production of a broad spectrum of products in *E. coli.* By enabling online controllability of protein expression, we believe that expression tuning is able to tackle the issues of constant product quality and culture long-term stability and therefore will pave the way for continuous production of biopharmaceuticals. This in turn will further progress *E. coli* as the primary expression platform for recombinant products intended for pharmaceutical or technical use.
